# Supplementary material for: GNAQ Q209R Mutations Are Highly Specific for Circumscribed Choroidal Hemangioma
Source: Cancers (Basel). 2019 Jul 22;11(7):1031. doi: 10.3390/cancers11071031 (PMC6679048; doi:10.3390/cancers11071031)
Supplement: Supplementary file 1 [file cancers-11-01031-s001.pdf]

GNAQ Q209R Mutations Are Highly Specific for Circumscribed Choroidal Hemangioma

Supplementary Material

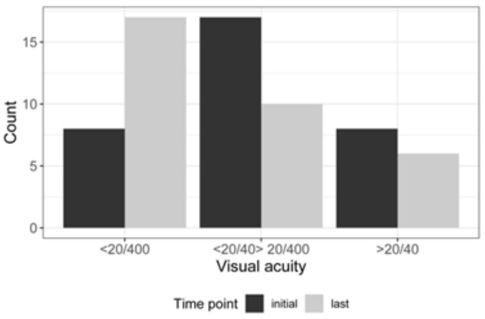

Figure S1. Visual acuity at initial (black) or last presentation (grey). Patients are grouped in three different groups: <20/400, visual acuity between 20/400 and <20/40 and >20/40.

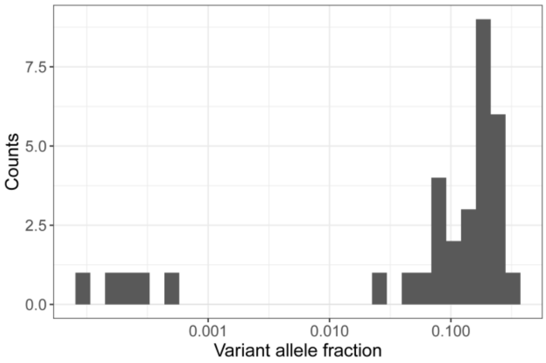

Figure S2. Distribution of the variant allele fractions over the CCH samples (n = 33). Y-axis shows the number of samples with the respective VAF. In five CCH samples, the VAF is below 0.001, which is regarded as background noise.

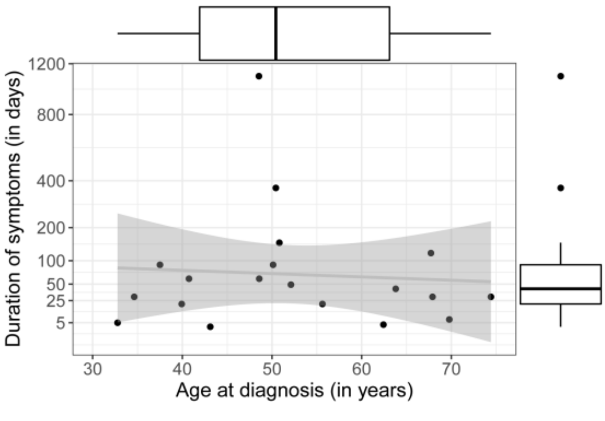

Figure S3. Dot plot showing relationship of age at diagnosis vs. duration of symptoms in mutation-positive CCH patients. *X*-axes shows age at diagnosis in years for each mutation positive individual, and duration of symptoms is shown on the *y*-axes (log-scale). Box plots are shown above/on the right side of the graph with the median as a solid line, and rectangles show the interquartile range and whiskers.

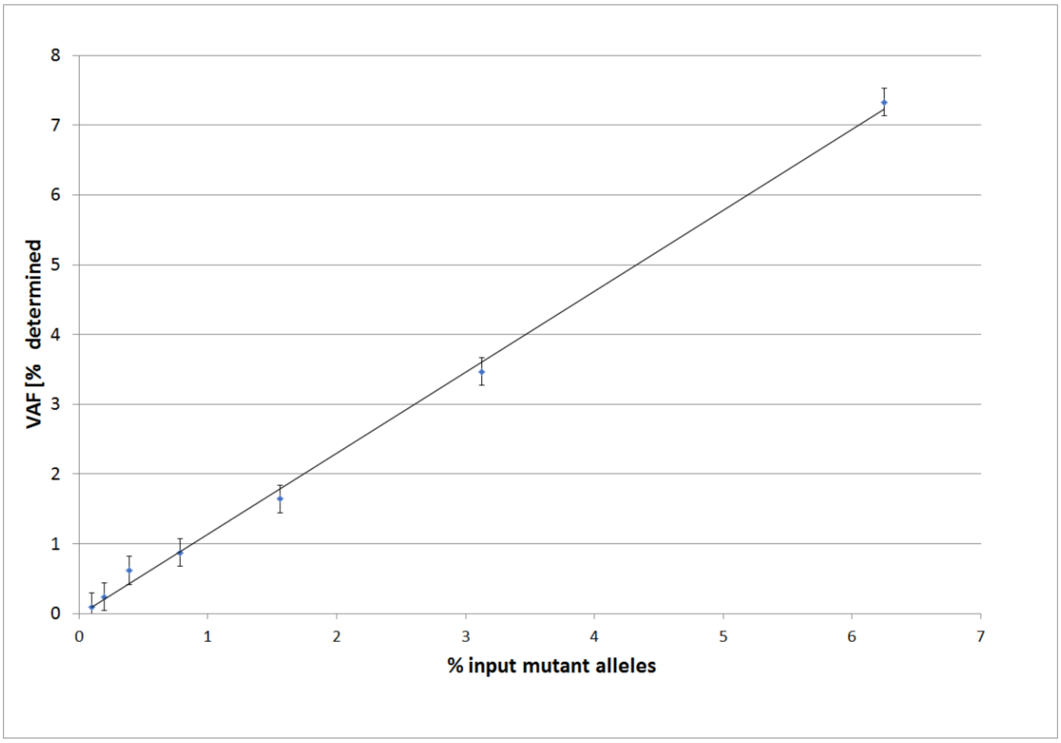

Figure S4. Standard curve: VAF [%], Variant allele fraction of mutant allele as determined by deep amplicon sequencing. Input % mutant alleles, different allele fractions were produced via the mixture of tumor DNA heterozygous for a *GNAQ* c.626A>T mutation with normal DNA. The mean VAF is given as calculated from duplicate and triplicate measurements which were performed for VAF > 0.5 and <0.5, respectively.

Table S1. Oligonucleotides for targeted amplification and deep amplicon sequencing on Illumina MiSeq used in first and second round PCR. Target specific sequence is given in small letters, Tag sequences are indicated in italic and Identifiers (ID) are given in bold. Platform (Illumina) specific adapter sequence is given in normal letters. For multiplex sequencing runs various ID sequences are used.

PCR	locus/name	Sequence 5' → 3'
1st round PCR Primers	GNAQ Q209fw	*CTTGCTTCCTGGCACGAG* atgatagaggtgacattttcaaagc
	GNAQ Q209rev	*CAGGAAACAGCTATGAC* aatatgagtattgttaaccttgcagaa
	GNAQ R183fw	*CTTGCTTCCTGGCACGAG* acttggaccgcgtagctg
	GNAQ R183rev	*CAGGAAACAGCTATGAC* aagtcaaaggggtattcgatga
	GNA11 Q209fw	*CTTGCTTCCTGGCACGAG* tgtgtcctttcaggatggtg
	GNA11 Q209rev	*CAGGAAACAGCTATGAC* ctgagggcgacgagaaacatgatgg
	GNA11 R183fw	*CTTGCTTCCTGGCACGAG* atcgccaccttgggctac
	GNA11 R183rev	*CAGGAAACAGCTATGAC* tgatgttctccaggtcgaaa
	GNAQQ209longfw	*CTTGCTTCCTGGCACGAG* gtgttacccagaatgttttaact
	GNAQQ209longrev	*CAGGAAACAGCTATGAC* tgcctgtctaaagaacacttac
	GNA14R205fw	*CTTGCTTCCTGGCACGAG* tccaagagcactggtttcca
	GNA14R205rev	*CAGGAAACAGCTATGAC* ccagcatacctcgttgtcac
	GNAQG48fw	*CTTGCTTCCTGGCACGAG* atggcactgtggtctgatga
	GNAQG48rev	CAGGAAACAGCTATGACTTGAGTGTGTCCATGGCTCT
2nd round PCR Primers	i5NSE501Ftag:	AATGATACGGCGACCACCGAGATCTACAC**TAGATCGC**ACACTCTTTCCCTA CACGACGCTCTTCCGAT*CTTATAGCCTTGCTTCCTGGCACGAG*
	i7N701Rtag:	CAAGCAGAAGACGGCATACGAGAT**TCGCCTTA**GTGACTGGAGTTCAGACG TGTGCTCTTCCGATCT*CAGGAAACAGCTATGAC*

mutation	GNAQ R183				GNAQ Q209				GNA11 R183		GNA11 Q209					References
Tumor	R183Q	R183G	R183L	R183C	Q209L	Q209P	Q209R	Q209H	R183C	R183S	Q209A	Q209L	Q209P	Q209R	Q209H	
AnastHema	0	0	0	0	1	0	0	8	0	0	0	0	0	0	0	26
BlueNev	0	0	0	0	7	0	0	0	0	0	0	0	0	0	0	12,16
CapilMal	8	1	1	0	0	0	0	0	3	0	0	0	0	0	0	6,11
CCH	0	0	0	0	0	0	34	0	0	0	0	0	0	0	0	13, this paper
CherryAngio	0	1	0	0	0	0	1	2	0	0	0	0	0	0	1	14
ChorNev	1	0	0	0	12	10	0	1	0	0	0	18	0	0	1	13,29, cosmic
CongenHema	0	0	0	0	10	4	0	2	0	0	0	8	0	0	0	10
DiffChHema	1	0	0	1	0	0	0	0	0	0	0	0	0	0	0	13, 31
forme_fruste_SWS	4	0	0	0	0	0	0	0	0	0	0	0	0	0	0	32
Hepatic_small_vessel_npl	0	0	0	0	0	0	0	2	0	0	0	0	0	0	0	28
leptomenigeal_melanocytoma	0	0	0	0	13	7	0	0	0	0	0	3	0	0	0	15,3
Melanocytoma	1	0	0	0	2	0	0	0	0	0	0	0	1	0	0	13,17
Melanoma_eye	19	0	0	0	164	242	8	6	20	0	1	378	5	1	6	cosmic
Melanoma_skin	2	0	0	0	23	14	0	1	6	0	0	28	0	5	1	cosmic
Nevus_skin	0	0	0	0	119	8	1	1	0	0	0	10	0	0	1	cosmic
PhakoPigmenVas	3	0	0	0	0	1	0	0	3	1	0	0	0	0	0	33
Pigm_Epithelioid_Melanocytoma	0	0	0	0	1	0	0	0	0	0	0	0	0	0	0	27
portwine_macrocheilia	19	0	0	0	0	0	0	0	0	0	0	0	0	0	0	20
Portwine_stain	29	2	0	3	0	0	0	0	0	0	0	0	0	0	0	13,19,22,38,39
portwine_stains_SWS	23	0	0	0	0	0	0	0	0	0	0	0	0	0	0	22
SWS	16	0	0	0	0	0	0	0	0	0	0	0	0	0	0	21,23

Table S2: Number of samples with *GNAQ* /*GNA11* mutations in different tumor entities.

References

38. Cai, R.; Gu, H.; Liu, F.; Wang, L.; Zeng, X.; Yu, W.; Zhang, X.; Liu, Y.; Ma, G.; Lin, X. Novel GNAQ mutation(R183G) of Port-wine stains: First case in East Asia. *Int. J. Derm.* **2019**, *58*, e75–e77.
39. Tan, W.; Nadora, D.M.; Gao, L.; Wang, G.; Mihm, M.C., Jr.; Nelson, J.S. The somatic GNAQ mutation (R183Q) is primarily located within the blood vessels of port wine stains. *J. Am. Acad Derm.* **2016**, *74*, 380–383.
